# Is medicines parallel trade ‘regulatory arbitrage’?

**DOI:** 10.1007/s10754-016-9199-z

**Published:** 2016-09-23

**Authors:** Joan Costa-Font

**Affiliations:** Department of Social Policy, London School of Economics and Political Science (LSE), Houghton Street, London, WC2A 2AE UK

**Keywords:** Parallel trade, Parallel imports, Regulatory arbitrage, Pharmaceuticals, Supply chain, I18, L51

## Abstract

Parallel trade (PT) is a phenomenon that takes place at the distribution level, when a patented product is diverted from the official distribution chain to another one where it competes as a parallel distributor. Although some research regards PT in Europe as a ‘common’ form of arbitrage, there are reasons to believe that it is a type of ‘regulatory arbitrage’ that does not necessarily produce equivalent welfare effects. We draw upon a unique dataset that contains source country records of parallel imported medicine sales to the Netherlands for one therapeutic group (statins), that accounts for 5 % of the market at the time of study and it faced no generic competition. We estimate precise differences in prices and statutory distribution margins for each source country/product and, examine whether they drive parallel import flows using a gravity specification and an instrumental variable strategy. Our findings reveal that parallel imports are driven by cross-country differences in statutory distribution margins in addition to price differences, consistently with the hypothesis of PT being a type of ‘regulatory arbitrage’.

## Introduction

In the European Union, medicines are regulated products subject to both single market and country specific health care regulations (e.g., medicines pricing and distribution regulation). This gives rise to parallel trade (PT), a phenomenon that takes place when a patented product is diverted from the official distribution chain (the distribution channel chosen by the originator) to another one in another European member state where it competes with the official distribution chain as a parallel distributor. PT is a legal activity even though medicines are products under the protection of intellectual property rights (IPR). This is the case because such IPR are subject to European-wide (as opposed to country wide) legal exhaustion after first sale in an existing European member state. A patent does not confer legitimate control of the product to the originator company upon sale in one-member state country. Hence, if price differences arise across countries, a parallel distribution chain may well be developed in higher price countries in response.

The consolidation of a single European market uncovers several opportunities for different forms of arbitrage. Typically, ‘common arbitrage’ takes place when an agent profits from product price differences across markets, which is expected to give rise to some form of price equalisation between markets, namely a common price. However, when price differences result from heterogeneous competitive conditions created from regulated prices (Costa-Font et al. 2014, 2015), as well as country specific statutory margins, we refer to such a phenomenon as ‘regulatory arbitrage’. In such a case, price equalisation is not guaranteed^1^, as regulations result from country specific lobbying and pressure group capacities (Grossman and Lai 2006).

A number of decisions adopted by the European Court of Justice have further encouraged distributors to engage in parallel trade (Barfield and Groombridge 1998). However, this paper does not attempt to examine the effects of parallel trade legislation, nor offer a state of the art of European parallel trade policy. Instead, we attempt to contribute to the literature by providing a different interpretation of how European PT should be regarded from an economics standpoint. More specifically, we examine here the extent to which parallel trade (PT) flows are driven by distribution chain regulations (and especially country specific statutory margins) rather than other factors including (unregulated) price differences which would drive more ‘common forms of arbitrage’ (Mauleg and Schwartz 1994; Richardson 2002; Jelovac and Borodoy 2005; Pecorino 2002). ‘Clean evidence’ that establishes the impact of distribution regulation is rare insofar as it requires access to source country data, and merge it with data on country specific statutory distribution margins (determining the profitability of the parallel trade business)^2^.

We empirically document whether European medicines parallel trade (PT) falls, at least partially, in the ‘regulatory arbitrage’ category. We add to the literature by taking advantage of a unique proprietary dataset (Intercontinental Medical Statistics, IMS) containing information on the source country of PT flows. Thus, we can compute for each product in the therapeutic group the exact price difference at the point of distribution. In addition, by identifying the destination and source countries, it is possible to match for each product/country the statutory distribution margins (that are added to the price^3^, which in turn provides a conservative estimate of the potential gain from PT given the potential presence of rebates and discounts which are not observed). We use data on parallel imports for cholesterol drugs from as many as eight European source countries (Belgium, France, Germany, Greece, Italy, Portugal, Spain, and the UK), which in our dataset are found to distribute up to 95 % of all observable parallel imported statins to the Netherlands across 24 quarters (1997–2002).

Previous studies have either failed to precisely identify the product origin, or have not taken into account the presence of generic drug penetration (Kanavos et al. 2008; Costa-Font et al. 2014, 2015). Furthermore, the theoretical literature remains inconclusive about the capacity of parallel trade to increase the country’s welfare (Mauleg and Schwartz 1994; Richardson 2002). That is, the normative implications for welfare of parallel trade expansion are ambiguous and tightly dependent on the welfare effects of a common price as compared to price discrimination equilibrium. Furthermore, previous empirical studies are limited by the wide therapeutic heterogeneity. This paper overcomes some of the limitations of previous studies.

Unlike previous empirical studies, our estimates are from a flexible augmented gravity specification of trade (import) flows that contains rich information on the heterogeneity of the supply chain regulation (more specifically retail and wholesale regulation). We can distinguish distribution chain regulation effects and we are able to estimate the volume of PT medicines in one country (the Netherlands) for a given therapeutic group from a given quarter. PT flows are modelled as a function of relative product prices (consistently with any form of arbitrage) and country specific wholesale regulations (specific of regulatory arbitrage), exchange rates (which is a variable that we separate from relative price to capture the adoption of a common currency in the period), and other covariates that are commonly employed in gravity specifications of trade such as geographical distance, and three different transformations of GDP (difference in GDP per capita, sum of GDP, relative GDP).

Our findings are consistent with the hypothesis of medicines’ parallel trade (imports) being an economic activity driven by cross-country differences in statutory distribution margins . An extension of the statutory margins can act as an incentive for PT even after controlling by medicines prices. The latter is (suggestive of and) consistent with the hypothesis of regulatory arbitrage as statutory distribution margins are determined by country regulation, which is specific of each country.

The remainder of the paper is as follows. In “Background” section provides the paper background. In “Data and Empirical Strategy” section presents the methodology, data sources and empirical strategy, while “Results” section reports the results and discusses them. Finally, “Conclusion” section outlines the main conclusions.

## Background

### Conceptual considerations

While PT is conceptualised in the theoretical literate as a ‘common’ form of arbitrage, predictions of arbitrage theory do not seem to be backed by empirical evidence (Kanavos and Costa-Font 2005). One explanation lies in the potential for some accommodative market equilibrium whereby drug companies accept a certain degree of PT (Ganslandt and Maskus 2004). Alternative explanations rest on the role of incentives resulting from country-specific regulations, and more specifically heterogeneous price regulations across countries in a single market such as the European one. In the pharmaceuticals sector, and despite Europe being an integrated market, regulatory interventions continue to take place at the national level, which applies both to the product price and to distribution margins (Kanavos and Costa-Font 2005). However, for arbitrage to take place, the market size of the source country needs should suffice to carry on the activity without leading to major shortages in the source country. Hence, the larger a particular market, the more attractive it is for both pharmaceutical manufacturers and parallel distributors to undertake production and trade respectively.

In most European countries, medicines are funded by a single payer (national health insurance or social insurance fund) who negotiates rates and purchases drugs on the countries behalf.$^{.}$ Hence, medicine prices in each country are not the result of market mechanisms, but some form of price regulation and bargaining. In addition, medicines are subject to regulation setting statutory distribution margins across Europe, and the most widely used model of distribution involves the manufacturer selling to the wholesaler and the latter to a retailer (pharmacy) with different degrees of vertical integration across countries. Assuming that payers regulate medicine prices as in Pecorino (2002) then, *ceteris paribus*, the larger the country market size, the higher the potential bargaining power of the health insurers (payers).

Manufacturers may follow a dual strategy in this case: they can either *deter* parallel imports by setting a sufficiently low (high) price in a high (low) price country such that it would make it unprofitable to perform parallel imports; or, alternatively, they can *accommodate* parallel trade simply by allowing parallel distribution to take place without necessarily taking action on prices. When arbitrage is unlimited then deterrence is more profitable than accommodation. Conversely, accommodation emerges when the potential volume of arbitrage is small and trade costs are relatively high (Ganslandt and Maskus 2004)^4^. However, the distribution chain regulation is not typically taken into consideration in previous studies despite PT is an activity that takes place at the distribution level.

### An empirical specification

Parallel imports can be empirically modelled, as any form of cross-country flow, using a gravity specification. The latter is widely used as a baseline model for estimating the impact of a variety of policy issues related to regional trading groups, currency unions and various trade distortions (Bougheas et al. 1999; Glink and Rose 2002). Gravity specifications can be expanded to incorporate both demand and supply factors specific of the pharmaceutical industry. A basic gravity specification would assume that a flow of goods (e.g., parallel traded medicines in our case) between two locations is *positively* related *ceteris paribus* to the size of the market (and income levels) and *negatively* related to the distance between them^5^. In our exercise, rather than structurally estimating the parameters of the gravity model, we aim at testing (in a reduced form) the significance of the parameters that are typically associated with trade flows, as well as those driving both common and regulatory arbitrage.

We assume parallel distributors aim at maximising an expected profit function, and hence are more likely to source products to both high price and high statutory distribution margin countries relative to the prices and distribution margins of the source countries. Given that the relevant price for parallel imported medicines is the wholesale price prevailing in any of the countries in question, parallel import prices depend, on a number of parameters related to drug distributors. The first is the nature of competition prevailing in the wholesale distribution business, which can be proxied by the number of wholesalers. Therefore, the research question our empirical strategy tries to address is that of empirically ascertaining whether parallel trade is any different from common arbitrage. More specifically, we will examine whether parallel imports are determined by the difference in wholesale margins across countries.

Our empirical strategy departs from a naïve pooled cross-sectional specification (OLS) and then expands it by taking into account the endogeneous determination of parallel imports and prices (2SLS). Then, we employ panel data techniques to offer a more robust specification that controls for potential unobservables that explain the correlation between observations for the same country (2SGLS). Our empirical strategy raises a number of econometric issues: namely, the inclusion of specific product fixed effects, given that gravity models do not typically allow for them. The second challenge lies in the limited variability in the regulation of medicines margins across time. To account for such effects we explore both pool and panel data specification possibilities. An augmented logarithmic version of the traditional gravity equation includes a number of controls as follows:

1 $$\begin{aligned} \log \left( {M_{ijt} } \right)= & {} \beta _0 +\beta _1 \log \left( {\frac{P_i }{P_j }} \right) +\beta _2 \tau _{ij} +\beta _3 \left( {Z_{ijt} } \right) +\beta _4 X_{ijt} +\beta _5 \zeta _{ijt} +\varepsilon _{ijt}\nonumber \\ Z_{ijt}= & {} \left[ \gamma _1 \log \left( {\left( {GDP_{it} +GDP_{jt} } \right) } \right) +\gamma _2 \log \left( {\left( {Y_{it} -Y_{jt} } \right) } \right) \right. \nonumber \\&+\gamma _3 (1-\left. \left( \frac{GDP_{it} }{GDP_{it} +GDP_{it}}\right) ^{2}\_\left( \frac{GDP_{jt} }{GDP_{it} +GDP_{it}}\right) ^{2} \right] \nonumber \\ X_{ijt}= & {} \left[ {\theta _1 \log \left( {\left( {Q_{it} +Q_{jt} } \right) } \right) +\theta _2 \log \left( {\left( {\frac{w_i }{w_j }} \right) } \right) } \right] \end{aligned}$$

where *i* and *j* denote origin and destination country respectively. The error term $\varepsilon _{ij}$ captures any other unobserved features that may affect bilateral trade between the two countries. Gravity-specific determinants are geographical distance $(\tau _{ij})$ the exchange rate $(\zeta _{ijt})$ and a number of other determinants $\left( {Z_{ijt} } \right)$ including bilateral sum of GDP of the two trading countries, the difference between GDP per capita of the two trading countries $\left( {Y_i -Y_j } \right)$, the relative country size. Given that parallel trade is theoretically conceptualised as a specific type of arbitrage (Ganslandt and Maskus 2004), it is arguably driven by the existence of a difference in relative prices between the two countries $\frac{P_i }{P_j }$. Finally, a number of key controls are included in $X_{ij}$. These include the relative margins of wholesalers in country *i* with respect to country $j (\frac{w_i }{w_j })$ and a volume effect in the form of total drugs from the specific therapeutic group of interest $Q_{it} +Q_{jt}$. Finally, $\beta$ denotes the vector of coefficients for each variable and $\varepsilon _{ij}$ measures the set of other influences on bilateral parallel imports which are part of the error term. Table 2 contains the lits of variables.

**Fig. 1:**
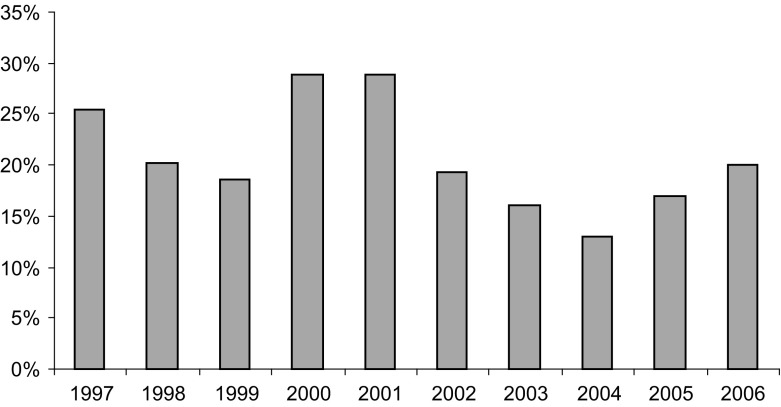
Market share of parallel imported statins in the Netherlands, 1997–2006. *Note* The figure reports the proportion of total parallel trade volume as a proportion of total volume of statins in the Netherlands for the period 1997–2002 (where we observed the origin of parallel trade) and 2003–2006 (where we only observe the the total volume but we could not identify the origin).it Source: The authors from IMS (2004)

## Data and empirical strategy

We use data from the Intercontinental Medical Statistics (IMS) database, which contain quarterly records on import sales and units over the 1997–2002 periods for a set of products that fall in the therapeutic product category of statins and exhibit parallel trade during the study period. The gives rise to a total sample size of 768 observations. However, due to the presence of some missing observations we were left with a final full sample of 625 for all variables. The data exhibits a three-way panel structure, 4 products 8 exporters to the Netherlands, in 24 quarters. Data for each product were made available at dispensation level. IMS collect data on prices and sales for a number of countries, including the Netherlands, and for the selected product group, statins, on a product-by-product (e.g. simvastatin, pravastatin, etc) and product presentation basis (e.g. simvastatin, 20 mg, 28 tablets). The accuracy of the data sources has been validated externally (IMS 2004). Data on both wholesale margin and regulation was obtained from publicly available sources for each country Ministry of Health. Rather than relying in different therapeutic groups, which are subject to different degrees of competition and the presence of generic competitors, we instead rely on a single therapeutic group (statins) which accounts for a significant proportion of total retail sales of prescription only medicines in European countries (5.7 % in 2002) (see Fig. 1).

Statins are products that lower levels of LDL (“bad”) cholesterol by 30–50 %, and have been popularly prescribed to prevent coronary heart disease (CHD), including myocardial infarction (MI), and their use has been increasing over time, making them, in turn, desirable targets for parallel trade. All products within the group were protected by a patent during the study period, therefore, the effect of parallel trade could be isolated from other effects, such as competition from generic equivalents, and studied without having to account for the competition effect due to generic penetration (Frank and Salkever 1991; Grabowski and Vernon 1992; Ganslandt and Maskus 2004).

Table 1 illustrates the difference in statutory margins and competition (number of companies) in the product distribution of each country. We report the number of competitors in the source country used to instrument distribution prices, as it is unlikely to affect the quantity of imports in any other way but though its effect on product prices. This is important for our instrumentation strategy below. Overall, Table 1 suggests marked differences in the regulation of prices and the wholesaling competitive conditions across European countries. We find that in France, wholesaler margins are the lowest in the period of analysis, which is consistent with France being the main source country of parallel imported medicines to the Netherlands. Similarly, we find Southern European countries exhibit a significantly higher fragmentation in their wholesaling and retailing practices compared to other European countries.

**Table 1:** Pharmaceutical price structure and distribution chain market structure in selected EU countries, 1997–2002. *Sources*: Paterson et al. (2003) and European Association of Pharmaceutical Wholesalers (2005)

Country	$\hbox {Ex-Manufacturer}^{2}$ (% price)	Number of wholesalers	Wholesaler margin $(\%\hbox { price})^{3}$	Pharmacy density (population per pharmacy)	Pharmacy margin $(\%\hbox { price})^{3}$
Netherlands	63.4	4	10.8	6100	20.2
Belgium	56.6	13	8.5	5200	29.2
France	64.8	$12^{1}$	3.8	2800	26.2
Germany	51.2	16	7.7	3900	27.3
Greece	63.1	130	5.5	1420	24
Italy	63.8	$95^{1}$	6.7	3700	20.4
Portugal	67.8	18	8.4	4000	19
Spain	62.7	51	6.7	2000	26.8

This table provides (for a number of countries of the study which are Medicine parallel exporters in to the Netherlands and the Netherlands) information on the average ex-factory (ex-manufacturer) price, the distribution margin of both distributors and manufacturers in such countries, and the average number of wholesalers and pharmacists in the countries of analysis

$^{1}$ Excluding regional offices and counting only head offices of the same wholesaler.

$^{2}$ Ex-manufacturer price as a proportion of price, assuming price = 100.

$^{3}$ Margins expressed as a proportion of price, assuming price = 100

**Table 2:** Variables and descriptive statistics

Variable	Abbreviation	Description	Mean
			(SE)
Dependent variable			
$\log \left( {M_{ijt} } \right)$	lquantity	Bilateral trade flow volumes of statins (logs) $^{\mathrm{a}}$	1.513
			(2.646)
Entry	Entry	Dummy variable measuring the entry of a new drug in the parallel trade market$^{\mathrm{a}}$	0.283
			(0.450)
(a) Gravity model controls			
$\tau _{ij}$	Ldist	Euclidean distance of latitude and longitude (in logs) of the country capitals	6.467
			(0.941)
$\log \left( {\left( {GDP_{it} +GDP_{jt} } \right) } \right)$	lG	Bilateral sum of GDPs (logs)$^{\mathrm{b}}$	10.782
			(0.107)
(b) Trade specification controls			
$\zeta _{ijt}$	Ler	Exchange rates in euros (logs)$^{\mathrm{b}}$	0.0679
			(0.102)
$\log \left( {Y_{it} -Y_{jt} } \right)$	labsR	Difference of per capita GDPs (absolute terms and logs)$^{\mathrm{b}}$ (N $=$ population)	0.949
			(0.618)
(c) Demand and capacity controls			
$\log \left( {\left( {Q_{it} +Q_{jt} } \right) } \right)$	lsumst	Sum of total sales of statins (logs)$^{\mathrm{a}}$	11.494
			(0.608)
$\mathbf{1}-\left( {\frac{{\varvec{GDP}}_{{\varvec{it}}} }{{\varvec{GDP}}_{{\varvec{it}}} +{\varvec{GDP}}_{{\varvec{it}}}}} \right) ^{{\varvec{2}}}\_\left( {\frac{{\varvec{GDP}}_{{\varvec{jt}}} }{{\varvec{GDP}}_{{\varvec{it}}} +{\varvec{GDP}}_{{\varvec{it}}}}}\right) ^{{\varvec{2}}}$	lS	Relative country size (logs)$^{\mathrm{b}}$	$-$0.711
			(0.247)
Treatment variables			
$\log \left( {\frac{P_i }{P_j }} \right)$	lrelP	Relative price between Netherlands and source country adjusted by defined daily doses (DDD)$^{\mathrm{a}}$	$-$0.359
			(0.436)
$\log \left( {\left( {\frac{w_i }{w_j }} \right) } \right)$	lrelMWS	Relative statutory distribution margins (logs)$^{\mathrm{c}}$	$-$0.518
			(0.296)

This table provides the descriptive statistics of all the variables employed in the study. The two dependent variables refer to the volume of parallel imported Medicines and the probability of entry for each product and time. Our treatment variables include (i) relative official wholesale difference in (regulated) margins between importing and exporting country exclusive of informal rebates (‘regulated arbitrage’), and the (ii) product price difference between importing and exporting countries (‘common arbitrage’). Finally, we consider a number of controls that can be classified as follows: (a) those that derive from a gravity equation of trade such as distance and bilateral sum of GDPs which should decrease and increase respectively the probability of trade. (b) Relative country size which explains the capacity of being a parallel exporter without producing major product shortages. (c) The size of the statin market which measures the demand for statins overall (sum of total sales of statins). (d) Income differences across countries and exchange rates with the euro which would be expected to respectively increase and decrease respectively the volume of trade. Export Country (i), Import County (j) and time (t)

*Sources:* $^{\mathrm{a}}$ IMS data 1997–2002

$^{\mathrm{b}}$ OECD Economic Outlook data 1997–2002

$^{\mathrm{c}}$ EFPIA, several years (www.efpia.org)

An important feature to note is that we were able to identify the price and volume of each product at any point in time. The dependent variable is the logarithm of imports of statins into the Netherlands. First, we use the basic specification and consider the impact of core explanatory variables such as GDP, population and distance. Subsequently, in line with recent theoretical developments (Egger 2002), we include variables measuring the size of trading countries and other barriers that might explain the development of parallel trade such as distance and exchange rates.

Although the influence of price differences on parallel imports to the Netherlands is consistent with the presence of both the common and regulatory arbitrage; the significance of differences in statutory distribution margins provides cleaner evidence of ‘regulatory arbitrage’. The only downside is that, as reported elsewhere (Kanavos and Costa-Font 2005), some of the gains from parallel trade are invisible because of the incentive structures of different stakeholders are influenced by rebates and informal discounts.

We first report the results of a pooled cross-section specification purely for comparative purposes it as relies on implausible assumptions (e.g., the presence of unobserved heterogeneity resulting from unobserved characteristics related to bilateral trade relationships). Then, we show the estimates of a panel data specification including country-pair “individual” effects, which partially account for the clustered nature of the data and hence, captures some of the existing unobserved heterogeneity. The panel specification refers to a generalised least squares (GLS) model with random effects consistent with the gravity specification outlined above whereby some variables are country-specific (e.g. distance) and does not allow country fixed effects. The interpretation of such a specification is that a country would export different volume of the same product to two other countries, even if their GDPs are identical and they are equidistant from the exporting country. This is due to potential differences in drug regulation, which affect prices and margins, and hence gains from PT.

We then estimate both two stage least squares (2SLS) and two stage generalised least squares (2SGLS) models to account for the endogenenity of price differences and the panel structure of the data. To instrument price differences, we are able to observe the variability in the competitive conditions in the drug distribution in the source country which we do not expect it would affect the volume of parallel traded product though other mechanisms but prices. Specifically, we use the relative number of wholesalers as an instrument for product price at the wholesale level. Given that prices are regulated, one would not expect they would conflate with direct price regulation in some countries (e.g., some countries might have free drug pricing and regulate heavily the margins of pharmaceutical distributors). We test and confirm the existence of endogeneity in the price formation, and the statistical validity of the instrument with the common F-test.

The variables employed in the analysis are presented in Table 2 and are reported as follows: (a) the observed volume of each statin imported into the Netherlands from another EU country; (b) the distance between two countries defined as the Euclidean distance of latitude and longitude between country capitals; the reason for measuring distance in this way rests on the fact that kilometers are not necessarily a good approximation for transport costs given alternative and more direct ways of transportation (e.g. air travel); (c) exchange rate is an obvious determinant of parallel imports insofar as it impacts price transparency (given that not all countries examined are in the euro area and the period examined corresponds to before the euro was introduced), especially in the context of European integration.; (d) following the predictions of a gravity model, our model includes the bilateral sum of country GDPs (in logs) $\ln \left( {GDP_{it} +GDP_{jt} } \right)$ , as it is conventional in the literature we measure relative country size (in logs), the difference of GDP per capita (in logs), and the sum of statins sales in € (in logs) that is the specific therapeutic group in question which has been growing in size during the study period which were included after testing for colinearity in the regression; (e) furthermore, we consider the point of entry of a parallel imported drug or product presentation. As expected from a model of arbitrage, relative prices between countries (in logs) should be a key determinant, with a negative expected sign. Finally, (g) a set of variables has been added to measure the aggregate number of distributors, which accounts for the degree of competition in the distribution chain in both countries proxied by the relative number of wholesalers in the Netherlands and the exporting country and the (h) relative wholesaler and account for possible economic incentives for parallel trade which are exogenous proxies for regulations.

**Fig. 2:**
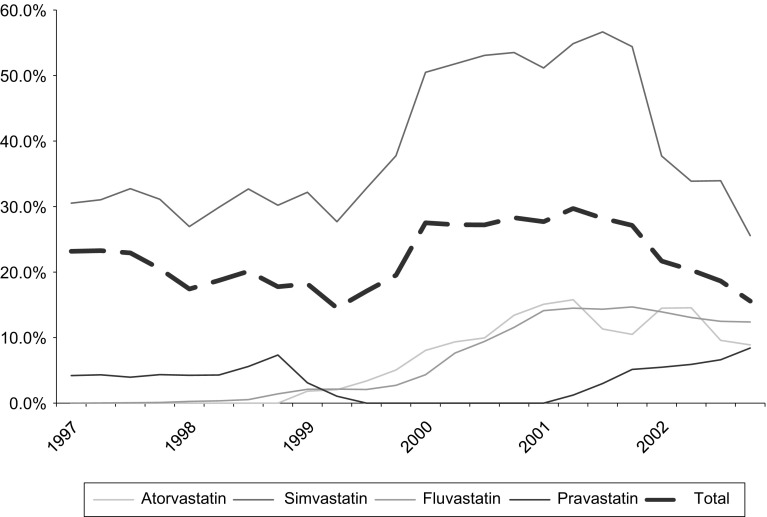
Parallel trade penetration of statins in the Netherlands (parallel imports as a % of total product market), 1997–2002. *Note* This figure reports (for each specific products that ate included in the statins therapeutic group) the penetration of parallel trade defined as the ratio of parallel traded volume and total volume in percentage terms. it Source: The authors from IMS (2004)

## Results

### Preliminary evidence

We begin our empirical examination by reporting descriptive evidence of trends in the parallel import penetration and establish some stylised facts that will guide our regression strategy. Specifically, Fig. 2 shows a monotonic penetration of parallel imported medicines to the Netherlands after 1999. Whilst this evidence is initially attributable to a single product (simvastatin), subsequently, it exhibits a comparable pattern for other competitor statins. According to IMS, the market shares of parallel imported statins were about 30 % over the study period, which suggest that the Netherlands is, compared to other European countries, one of the most dynamic parallel importers.

**Fig. 3:**
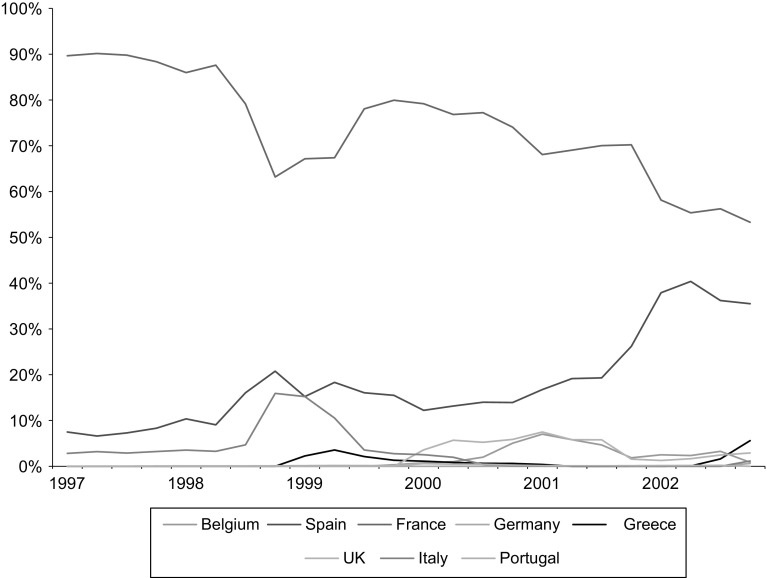
Proportional (%) origin of parallel imported statins in the Netherlands, 1997–2002. *Note* This figure displays for each year of the study 1997–2002 the proportion of parallel trade sales of statins in the Netherlands per country of origin. it Source: The authors from IMS (2004)

Figure 3 reports PT trends by country of origin. Importantly, the most common country of origin of parallel imported drugs in the Netherlands, at least in the earlier parts of the study period, was France. This is result cannot be explained by price differences alone given that France does not exhibit the lowest price among exporting countries. In contrast, France does exhibit the lowest statutory distribution margins in the period of analysis. In addition, the relative large size of the French market allows some distribution to be shifted without compromising too much medicines availability. Finally, and consistent with a gravity specification, France is, together with Belgium, the closest geographical neighbour to the Netherlands.

Importantly, the evidence presented in Fig. 3 suggests that although 90 % of parallel imported statins into the Netherlands were sourced from France in 1997, Spain’s market share has increased significantly since 2000. By 2002 Spanish exports accounted for 40 % of all statins parallel imported into the Netherlands.

**Table 3:** Augmented gravity equation estimates (OLS and 2SLS) dependent variable: bilateral parallel trade flows to the Netherlands (in $$M_{it})^{*}$$

	OLS (3.1)	2SLS (3.2)	2SGLS – RE (3.3)
	Coeff	Coeff	Coeff
	(SE)	(SE)	(SE)
lrelMWS	$-$0.969	$-$1.754$^{\mathrm{c}}$	$-$1.643$^{\mathrm{a}}$
	(0.719)	(0.935)	(0.536)
lrelP	$-$0.501	4.012$^{\mathrm{a}}$	3.458$^{\mathrm{b}}$
	(0.306)	(1.059)	(1.671)
ldist	1.809$^{\mathrm{a}}$	5.877$^{\mathrm{b}}$	6.564
	(0.263)	(2.939)	(4.951)
ler	$-$1.638	$-$7.042$^{\mathrm{b}}$	$-$2.225
	(1.683)	(3.068)	(9.129)
lG	$-$11.90$^{\mathrm{a}}$	$-$15.815$^{\mathrm{a}}$	12.690$^{\mathrm{a}}$
	(3.384)	(5.027)	(3.58)
lS	145.798$^{\mathrm{a}}$	193.058$^{\mathrm{a}}$	77.845$^{\mathrm{a}}$
	(18.978)	(34.457)	(23.38)
labsR	$-$4.278$^{\mathrm{a}}$	$-$5.067$^{\mathrm{a}}$	$-$1.176
	(0.742)	(1.091)	(1.255)
lsumst	3.164$^{\mathrm{a}}$	3.852$^{\mathrm{a}}$	$-$1.798$^{\mathrm{b}}$
	(0.638)	(0.896)	(0.717)
Intercept	188.991	245.661$^{\mathrm{a}}$	$-$78.49
	(40.584)	(62.091)	(37.979)
$\hbox {R}^{2}$ (adjusted)	0.15		
N (observations	625	610	610
Wald $\chi _8^2 \left( {\nabla \beta _i =0} \right)$		82.33	57.38

* Restricted to molecules where there is some evidence of parallel trade.

$^{\mathrm{a}}$ Denotes significance at 1 % level

$^{\mathrm{b}}$ Denotes significance at 5 % level, and estimates contain robust standard errors

### Econometric results

This section reports the econometric estimates of a number of gravity specifications following the premises of equation (1), which they will allow us to identify the influence of economic versus regulatory determinants of parallel import entry and penetration. Appendix 2 provides the definition of the variables employed and how they link to equation 1. Purely for comparative purposes, Table 3 provides the estimates of a pooled OLS specification, alongside with two stage least squares (2SLS) and two-stage generalised least squares (2SGLS) estimates. Given the panel nature of the data, we cluster our observations by both country of origin and product. Yet, as the observations are not independently clustered, our preferred specification is that of a GLS model that accounts for alternative distributional assumptions. The treatment variables in the specification refer to both the relative price differences between importing and exporting countries and the difference in regulatory margins which influence the wholesalers decision to source to other countries through PT. Accordingly, we include as a key covariate the statutory distribution margins difference to measure the effect of competitive conditions in the drug distribution system.

Most variables reveal the expected coefficients, specifically we divide the variables between treatment variables including statutory margins and price differences measuring the effect of common and regulatory arbitrage as defined in Table 2, and those variables that can be labelled as controls. The difference between different controls lies in that some are covariates specific of a gravity specification (distance and country mass), other are economic controls such as (income and exchange rates) which are commonly associated with trade volume, and finally we include other variables specific of the arbitrage activity (demand for statins proxied by the total market and the country size).

Endogeneity tests (Wooldridge 2010) suggest unambiguous evidence of endogeneity of price differences. (we can reject the hypothesis of exogeneity) Accordingly, we have instrumented the price differences using data on the relative number of wholesalers across countries. The theoretical justification for including these instruments lies in the fact that they are strongly associated with the formation of prices given that statutory margins are responsible for the formation of final prices but one would not expect them to influence volume directly. An instrumental variable (IV) estimation should provide a consistent estimate of the coefficients of interest and correct for any omitted variable bias (Angrist and Krueger 2001).

Estimates reported in Table 3 are for the most part consistent with expectations. We find that barriers to trade—such as exchange rates—exhibit the expected (negative) effects on parallel imports^6^. Table 3 reports the results of a gravity equation specification estimated using OLS, 2SLS and 2SGLS. The logic of the latter specification rests on the assumption that, country- and product-specific effects might be driving the dynamics of parallel import flows. Fixed effects would not be consistent with a model including distance so we rely on random effects. We considered estimating the model using country of origin fixed effects, however two of the most relevant variables could not then be included in the specification. Hence, we have decided not to pursue such strategy.

Table 3 suggests that some of the common determinants of trade do not apply to parallel imports. Indeed, transport costs might turn out to be less robust and not statistically significant. However, the estimate difference between 2SLS and OLS estimates with regards to the effect of relative prices and wholesale margins is suggestive of the presence of unobservables and other confounder effects biasing our results. Specifically, GLS estimates suggest that in addition to pure price differences (elasticity of 3.4 of a unit difference in relative prices), the effect of distribution margins remains significant consistently with the hypothesis of regulatory arbitrage. Our preferred specification is consistent with expectations with regards to the gravity model (positive effect of combined economic mass (GDP) but no effect of country distance), a higher demand for statins would reduce the volume of parallel imports whilst country size is expected to produce the opposite effects on trade. Table 4 in the Appendix provides comparable regression results to predict the entry in the parallel trade market, and suggests that the same regulatory variables explain the decision to enter the market.

An important picture comes out of such a strategy. Consistently with PT as a form of regulatory arbitrage, medicine price differences which (are set mainly by government regulation) exert a significant effect in explaining parallel trade volume, but a large proportion of parallel import flows are driven by changes statutory distribution margins between the Netherlands and source countries in line with the regulatory arbitrage hypothesis. The effect size of the difference in wholesale margin compares to that of a change in total volume of statins, which we use here to proxy product demand.

## Conclusion

We have empirically examined the hypothesis of PT being a type of regulatory arbitrage that takes place at the medicines distribution level. We have studied the combined influence of both distribution chain and price regulation in driving PT flows drawing from unique data (that includes source country information, and hence allows estimating the effect of both specific price differences and cross-country statutory margins). Our dataset is from the Netherlands (a country that ranks among the highest parallel importers) and for one therapeutic group: statins (a therapeutic group subject to patent protection during the period of analysis and not affected by generic entry). We specifically examine the influence of differences in both price and statutory margins between the Netherlands and country of origin countries in driving parallel trade flows.

Our results are consistent with the hypothesis of parallel trade in Europe being indeed a regulation-induced phenomenon, which we refer to as ‘regulatory arbitrage’. That is, we find that changes in the statuary margins across countries afford incentives for the development of parallel distribution channels. Hence, even if all European countries were not to regulate medicines prices, there could still be some potential for PT driven by differences in statutory distribution margins. Nonetheless, our results need to inevitably be taken with caution as they rely on only one therapeutic group, and refer to a period where the European Union was restricted to 15 members. One would expect differences in the nature of the activity in an enlarged Europe.

## Appendix 1

See Table 4.

**Table 4:** Entry (Probit) Dependent variable: 1 refer to the existence of some trade flows to the Netherlands (in $$M_{it})^{*}$$

	Entry (Probit)	
	Coeff	SE
Ldist		0.999$^{\mathrm{a}}$
		(0.148)
lrelP		0.386$^{\mathrm{b}}$
		(0.159)
Ler		$-$0.255
		(0.869)
lG		$-$3.663$^{\mathrm{b}}$
		(1.856)
lS		74.108$^{\mathrm{a}}$
		(12.383)
labsR		$-$2.209$^{\mathrm{a}}$
		(0.419)
Lsumst		1.223$^{\mathrm{a}}$
		(0.347)
lrelMWS		$-$0.458$^{\mathrm{b}}$
		(0.202)
Intercept		72.694
		(23.547)
$$\hbox {R}^{2}$$ (adjusted)		
N (No. of observations)	625	
Pseudo $\hbox {R}^{2}$	0.13	
Likelihood ratio $\chi _8^2$	91.29	

* Restricted to molecules where there is some evidence of parallel trade.

$^{\mathrm{a}}$ Denotes significance at 1 % level

$^{\mathrm{b}}$ Denotes significance at 5 % level, estimates contain robust standard errors

## Appendix 2: Variable description

Bilateral trade flow volume **lquantity**

Distance **ldist**

Exchange rate **ler**

Entry **entry**

**LabsR** . Referes to the difference of log of GDPpc between export and import country, representing a proxy for country’s relative factor endowment. The smaller the difference, the more intra-industry trade and the lower inter-industry trade. Expected sign: negative

$$\begin{aligned} R_{ijt} =\left| {\log \left( {\frac{\hbox {GDP}_{it} }{N_{it} }} \right) -\log \left( {\frac{\hbox {GDP}_{jt} }{N_{jt} }} \right) } \right| \end{aligned}$$

Following Egger (2000)

- **lG** is the Bilateral sum of GDP: the larger the overall economic space, the larger trade between these two countries. Expected sign: positive
  $$\begin{aligned} G_{ijt} =\log \left( {\hbox {GDP}_{it} +\hbox {GDP}_{jt} } \right) \end{aligned}$$
- **lS** is the Relative country size: the larger the measure, the more similar the two countries in terms of GDP, and therefore, the more intra-industry trade. Expected sign: positive
  $$\begin{aligned} S_{ijt} =\log \left( {1-\left( {\frac{\hbox {GDP}_{it} }{\hbox {GDP}_{it} +\hbox {GDP}_{jt} }} \right) ^{2}-\left( {\frac{\hbox {GDP}_{ji} }{\hbox {GDP}_{it} +\hbox {GDP}_{jt} }} \right) ^{2}} \right) \end{aligned}$$

**lsumst** refers to the Sum of total sales of statins (log). Expected sign: positive

$$\begin{aligned} \mathbf{ln}(\mathbf{Q}_{\mathbf{i}}+\mathbf{Q}_{\mathbf{j}}) \end{aligned}$$

The logic of a additive specification would be that the number of sales stand out as a proxy for market size.

**lrelWS** refers to the relative number of wholesalers as an IV for price difference. IV

Expected sign: positive

- We are interested in the relative number of wholesalers between the two countries, since the higher and positive the difference, the more parallel trade will take place.

**lrelP** is the relative price between Netherlands and source country. *Expected sign: negative*. A high ratio between pi and pj means that pi is much larger than pj, therefore, the higher the price in export country, the lower the parallel trade.

$$\begin{aligned} \mathbf{Ln}(\mathbf{p}_{\mathbf{i}}/\mathbf{p}_{\mathbf{j}}) \end{aligned}$$

**lrelMWS** is the relative price between WS margins in export and import country. *Expected sign: negative*. A high ratio between wholesalers margins export and import country means that wholesalers margin is much larger in export country, therefore, the higher the margin in export country, the lower the parallel trade.

Same logic as price difference.

## Footnotes

1 The term ‘regulatory arbitrage’ is not specific to the pharmaceutical industry regulation. Typically, in other industries, that term is applied when a bank changes its charter from a state bank to a national bank to take advantage of different regulatory treatment, or when a firm changes its country of headquarters to take advantage of different tax regimes.

2 Parallel imports may well be the effect of second degree price discrimination, for example resulting from discounts given by parallel distributors to pharmacists in importing countries (Anderson and Ginsburgh 1999).

3 This paper fills this gap by using a unique dataset, which contains a rich set of controls for the regulation of the distribution chain. This is especially important given that identifying bilateral trade flows allows us to ascertain the magnitude of the effect of economic arbitrage (price differences between country of origin and destination) *vis a vis* distribution chain regulatory differences.

4 Given that the distribution of medicines is heavily regulated across European countries, parallel imports might well result from the lack of total vertical control in the pharmaceutical distribution chain by the manufacturer.

5 A structural gravity model could arise from a CES preferences and increasing returns to scale. In that context, distance can be related to transport costs and operates like a relative price, with a coefficient (elasticity) of unity in most cases.  GDP (market size) variables are there to account for importer demand and exporter capacity and both should have positive coefficients.

6 This has to do with the fact that in some parallel exporter countries such as Spain and France the introduction of the euro has eliminated the exchange rate variability with the Netherlands
